# Comparison of the simulated outcomes of aerosol–cloud interaction by a meteorological model with and without an interactive chemistry module

**DOI:** 10.1038/s41598-023-32355-4

**Published:** 2023-04-07

**Authors:** Azusa Takeishi, Chien Wang

**Affiliations:** Laboratoire d’Aérologie, UPS/CNRS, 14 avenue Edouard Belin, 31400 Toulouse, France

**Keywords:** Atmospheric chemistry, Atmospheric dynamics

## Abstract

With the general rise of computational capacities and the continued efforts to improve computational efficiencies, more and more studies have been utilizing state-of-the-art atmospheric models that enable cloud-resolving simulations over a global domain. Microphysical processes inside clouds, however, are on a scale much smaller than that of a cloud itself, and therefore resolving clouds in a model is not equivalent to resolving cloud microphysical processes. When aerosol–cloud interaction (ACI) is studied, chemistry models enable the prognostic calculations for chemical species, including aerosols, which can perturb cloud microphysics and eventually impact clouds and climate. The large drawback of these models is the high computational cost required for tracking chemical species in space and time that may not be affordable in some studies. As a result, some studies have used non-chemistry models with prescribed cloud droplet number concentrations $n_c$ and compared multiple simulations with different $n_c$ to assess the impacts of varying aerosol concentrations on clouds. In this study we assess whether the same or similar ACI can be simulated when the aerosol number is increased in a chemistry model and when $n_c$ is raised in a non-chemistry model. A case study has been conducted over the Maritime Continent in September 2015 when an extremely large number of aerosols were observed due to fires under a dry condition brought by a strong El Niño. The comparison of the simulations by the chemistry and non-chemistry models shows that the aerosol-driven enhancement of rainfall in the chemistry simulations was not present in the non-chemistry simulations, even with prescribed $n_c$ with certain spatial variation based on the chemistry runs. Therefore, simulated ACI may largely differ depending on the manner by which an increase or decrease of aerosols is represented in a model. The result suggests the further need for powerful computational capabilities and the pursuit of a rigorous means to incorporate aerosol species in a non-chemistry model.

## Introduction

Aerosol–cloud interaction (ACI) has been an activate area of research over the past few decades, especially following the Intergovernmental Panel on Climate Change (IPCC) report that suggested ACI as one of the largest uncertainties in the climate system^1,2^. One of the primary reasons for such a large uncertainty is the complexity of aerosol–cloud microphysical processes (i.e., aerosol indirect effects) that cannot be easily represented in large-scale models. The role of aerosols as cloud condensation nuclei (CCN), for instance, depends heavily on the background aerosol conditions, which include number concentrations, size distributions, and types of aerosols that determine the hygroscopicity $\kappa$^3^. Chemistry models are able to track these variables concurrently with the abundance of gas-phase chemical species. On the other hand, many of the regional models do not include these chemistry or aerosol variables, since tracking them for a variety of aerosol types raises the required computational power significantly. Therefore, in order to assess the impacts of varying CCN concentrations on clouds and precipitation with these models, variations of aerosol concentrations are represented by those of cloud droplet number concentrations $n_c$, or sometimes a single prescribed $n_c$ in simulations; this idea stems from the fact that $n_c$ is generally proportional to ambient aerosol number concentrations $n_a$, and therefore, the fluctuations of $n_a$ can be roughly represented by increasing or decreasing $n_c$ in simulations. Although the comparisons of these simulations with prescribed $n_c$ give us great insights into how sensitive cloud processes are to CCN concentrations in a conceptual manner, it remains uncertain as to whether they accurately represent the realistic ACI if they lack the temporal (and maybe spatial) variations of $n_c$.

In this study, we compare the impacts of increased aerosols on clouds and precipitation, simulated in the Weather Research and Forecasting (WRF) model^4^ versus WRF coupled with Chemistry (WRF-CHEM)^5^; the latter explicitly calculates $n_c$ as a result of activation of ambient aerosols and other microphysical processes, while the former uses time-invariant prescribed $n_c$. The spatial patterns of this time-invariant $n_c$ in the WRF runs were obtained from the simulated $n_c$ in the WRF-CHEM simulations by temporally averaging the vertical maximum droplet number concentration in each column ${\overline{n}}_{zmax}$ (see “Methods” section for details). Therefore, $n_c$ in the WRF simulations are spatially variable but temporally unchanged for the whole simulation period. We conduct a month-long case study over the Maritime Continent in September 2015 where/when an extremely dry condition was caused by a strong El Niño, which resulted in enhanced emissions of biomass burning particles. According to Takeishi and Wang (2022; hereinafter TW22)^6^ in which WRF-CHEM simulations with and without fire particles (FIRE and NOFIRE runs, respectively) were run, the increased aerosol number led to an increased rainfall on a monthly average. In TW22, we showed that the increased $n_c$ or smaller cloud droplet populations in the FIRE run led to enhanced production of ice crystals and other frozen hydrometeors such as snow and graupel, which resulted in increased surface precipitation; the results of our analyses were consistent with the hypothesis that smaller cloud droplets, which remain longer in air without falling out, created favorable condition for more frozen hydrometeor formation/growth via more freezing into ice crystals aloft and/or more riming. This study runs a pair of WRF simulations, which is comparable to the one with WRF-CHEM in TW22, with low and high $n_c$ to represent cases with and without the presence of fire aerosols, respectively, and aims to answer the following question: *Does a change in cloud droplet number concentrations* $n_c$ *in WRF lead to the same ACI as in WRF-CHEM?* In the rest of the paper, WRF simulations with low and high $n_c$ are referred to as WRF-NOFIRE and WRF-FIRE, respectively, and the NOFIRE and FIRE runs in WRF-CHEM presented in TW22 are here referred to as CHEM-NOFIRE and CHEM-FIRE, respectively. Note that the primary focus of this study is the comparison of ACI (i.e., FIRE-NOFIRE) in WRF and WRF-CHEM, given the more accurate and realistic representations of aerosol–cloud interaction in WRF-CHEM simulations.

## Results

The results of the CHEM runs are already presented in TW22. In this paper, we focus on the “fire effect” in the new WRF simlations, which refers to the difference between FIRE and NOFIRE runs hereinafter.

**Figure 1:**
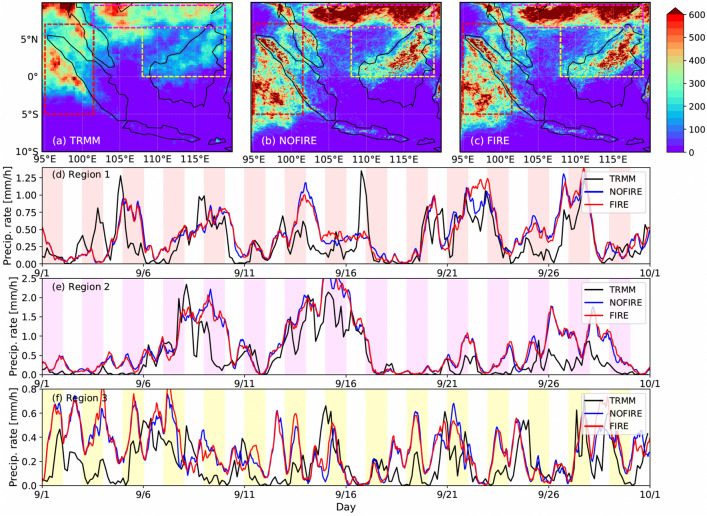
Accumulated precipitation (mm) for the month of September in 2015, (**a**) observed by TRMM and (**b**) simulated in WRF-NOFIRE and (**c**) WRF-FIRE. Red, magenta, and yellow rectangles show the locations of Region 1 (95 W–101.5 W, 5 S–7 N), Region 2 (101.5 W–119 W, 6.5 N–9.5 N), and Region 3 (108 W–119 W, 0–6.5 N), respectively. Time series of TRMM (black, 3-hourly) and simulated (blue and red, hourly) precipitation rates (mm/h), averaged over each region, are shown for (**d**) Region 1, (**e**) Region 2, and (**f**) Region 3. An equivalent figure for the CHEM runs can be found in TW22.

Figure 1 shows the observed and simulated spatial distributions of monthly accumulated rainfall (Fig. 1a–c) and the temporal evolution of surface rainfall (Fig. 1d–f) averaged within three major rainfall areas as in TW22. Although the general overestimation of the accumulated precipitation can be seen in our simulations (Fig. 1b,c) when compared to the estimates by the Tropical Rainfall Measuring Mission (TRMM^7^, Fig. 1a), the simulations capture the general patterns of spatial distributions and the temporal evolution; large amounts of surface precipitation are seen over west of Sumatra (Region 1; Fig. 1d), the southern part of the South China Sea (Region 2; Fig. 1e), and northern Borneo (Region 3; Fig. 1f). The temporal evolution is well captured especially in Regions 1 and 2 (Fig. 1d,e) where the large precipitation events are simulated at a right timing with slight overestimation of the rainfall rates. In addition, the differences in the time series of the three regions suggest that each of them experiences different precipitation systems at different frequencies. Indeed, Region 1 is mainly impacted by the Sumatra squall lines that bring moisture from the southwest, and Region 3 or Borneo typically exhibits a clear diurnal cycle of rainfall, as explained by Lee and Wang^8^. Region 2 experiences a second rainy season of the South China Sea in September, according to Hu et al.^9^. These events all have different frequencies, which explains the differences in the time series shown in Fig. 1d–f.

**Figure 2:**
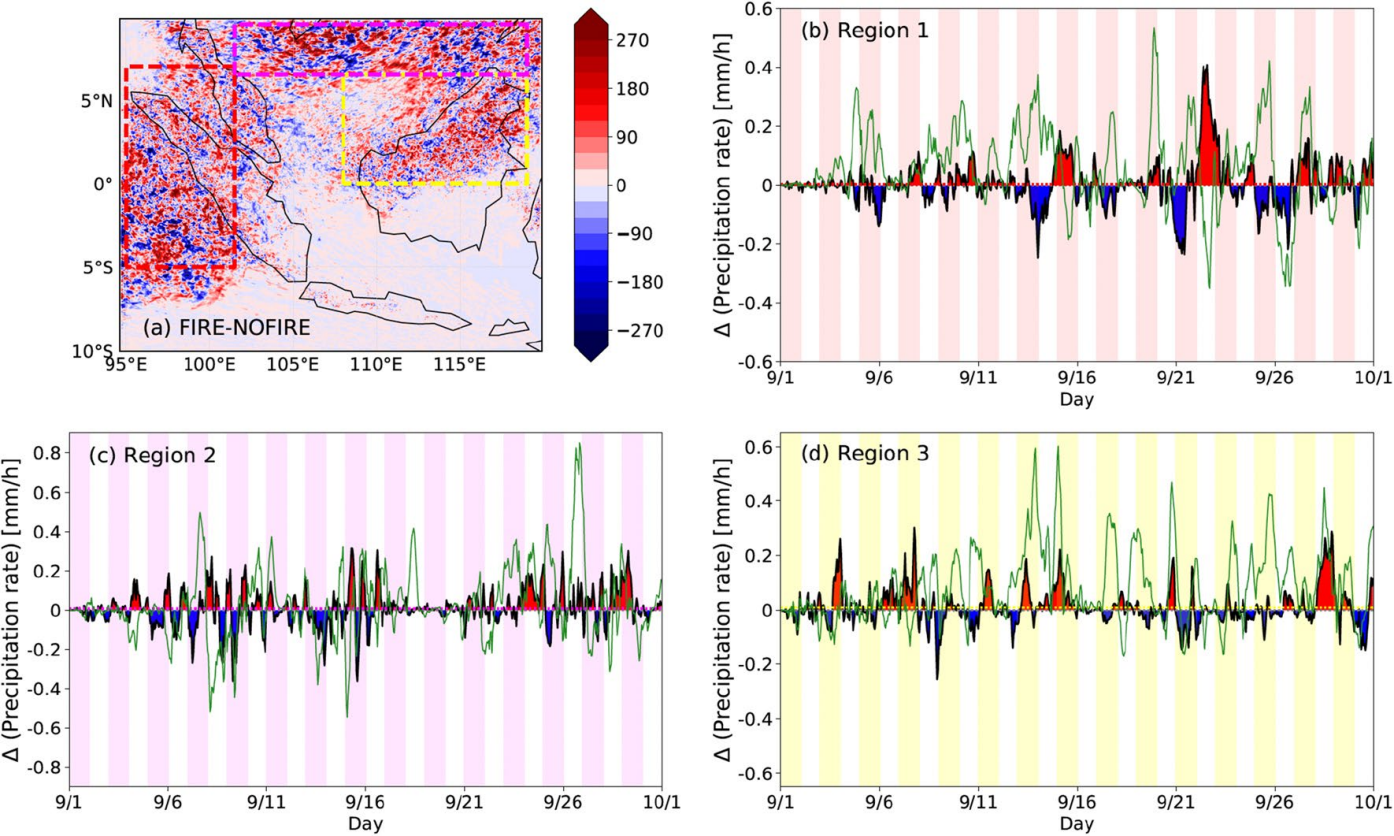
(**a**) Difference (FIRE-NOFIRE) in accumulated precipitation (mm) over the month of September. (**b**–**d**) Time series of regional mean precipitation rate differences (FIRE-NOFIRE) in (**b**) Region 1, (**c**) Region 2, and (**d**) Region 3. An equivalent figure for the CHEM runs can be found in TW22. The green lines show the equivalent values from the CHEM runs.

When it comes to the difference between WRF-FIRE and WRF-NOFIRE, however, it is very small; Fig. 2 shows the differences (FIRE-NOFIRE) in the spatial patterns of monthly accumulated surface rainfall (Fig. 2a) and the time series of precipitation rates in the three regions (Fig. 2b–d). In all the three regions, there are spots of a rainfall increase (red) and reduction (blue) but no clear sign of one or the other spatially dominating the region (Fig. 2a). Indeed, the FIRE-NOFIRE differences in area-mean monthly total rainfall (mm) is on the order of $10^0$ (mm), which is much smaller than the same differences between CHEM-NOFIRE and CHEM-FIRE that are largely positive and on the order of $10^1$ (mm) (Table 1). As for precipitation rates, the monthly mean differences (dotted lines in Fig. 2b–d) are extremely small, indicating the lack of consistent increase or decrease on a monthly average. The green lines in Fig. 2b–d show the differences between CHEM-FIRE and CHEM-NOFIRE, which show much larger values especially on the positive side (i.e., FIRE > NOFIRE). Table 1 lists the differences between the WRF runs and the CHEM runs, the former of which shows values that are about an order of magnitude smaller than the latter for both total rainfall and mean rainfall rates. Furthermore, Supplementary Figure S1 shows the monthly mean vertical profiles of hydrometeor mass mixing ratios, along with the frequency distributions of cloud liquid and ice water contents, estimated cloud optical thickness, and cloud top height in all the four simulations, averaged within Region 3 where the largest difference in rainfall was simulated. The vertical profiles confirm the insensitivity of the WRF runs compared to the CHEM runs that present largely increased snow and rain masses in CHEM-FIRE, as discussed in TW22. As for the two-dimensional cloud properties, the FIRE-NOFIRE differences are small even in the CHEM runs. This small difference may be due to the dynamical constraint on the tropopause height and moisture availability, as well as inherently high cloud optical thickness of convective clouds. Nonetheless, we still see subtle differences in these variables between CHEM-FIRE and CHEM-NOFIRE, which confirms the increased ice mass, increased cloud optical thickness (i.e., smaller droplets and increased cloud mass), and slightly increased cloud-top height in CHEM-FIRE under the presence of many fire particles. By comparing the WRF and CHEM simulations as such, the effects of including the full chemistry processes on rainfall have become evident. That is, in terms of monthly mean precipitation rates and monthly accumulated rainfall, the WRF simulations did not capture the fire effect that was seen in the CHEM simulations due to the lack of temporal and spatial variations of $n_c$ in the magnitude predicted by the full chemistry module.

**Table 1:** Differences (FIRE-NOFIRE) in monthly total surface rainfall (mm) and monthly mean rainfall rates (mm/h) averaged over each region.

	Total rainfall (mm)			Rainfall rate (mm/h)		
$\Delta$(FIRE-NOFIRE)	Region 1	Region 2	Region 3	Region 1	Region 2	Region 3
WRF	+ 2.55 (0.83$\%$)	+ 6.07 (1.17$\%$)	+ 7.17 (3.40$\%$)	+ 0.0035	+ 0.0084	+ 0.010
CHEM	+ 42.60 (17.15$\%$)	+ 18.16 (4.04$\%$)	+ 61.60 (34.51$\%$)	+0.059	+ 0.025	+ 0.086

The changes in percentages are in comparison to the monthly mean values in NOFIRE, and the same percentage applies to both total rainfall and rainfall rate in the same run.

**Figure 3:**
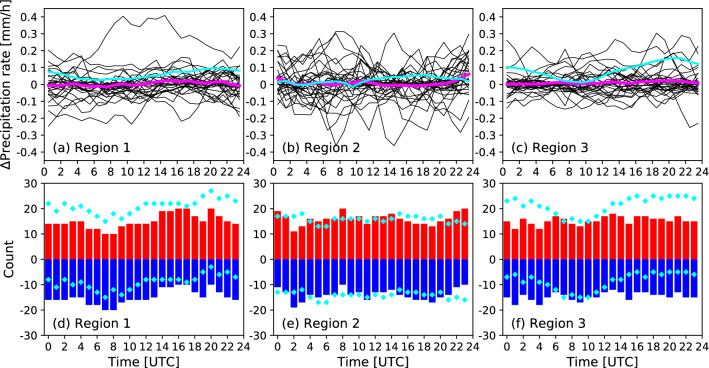
(**a**–**c**) FIRE-NOFIRE differences in hourly precipitation rate (mm h$^{-1}$) each day (black) and their monthly average (magenta). (**d**–**f**) Raw counts of increased (+ 1, red) or decreased (− 1, blue) hourly rainfall rates (FIRE-NOFIRE). All are averages over (**a**,**d**) Region 1, (**b**,**e**) Region 2, and (**c**,**f**) Region 3. An equivalent figure for the CHEM runs can be found in TW22. The cyan lines and markers present the equivalent data from the CHEM runs.

The amount of rainfall in our WRF simulations did not change as largely as in WRF-CHEM, but we further examine if we see any changes in the diurnal cycle of rainfall, which may be missed out in monthly total (Fig. 2a) or monthly mean rainfall rates (Fig. 2b–d). Figure 3a–c shows the differences in precipitation rates plotted separately for each day in the three regions, with a monthly average shown in magenta. All of the magenta lines stay nearly zero, which indicates the lack of consistent changes in diurnal cycle due to increased $n_c$ in WRF-FIRE. The same average for the CHEM runs (cyan lines), however, shows a slightly enhanced rainfall later in the day in UTC, which corresponds to early morning hours in local time. In TW22 we concluded that this enhanced morning rainfall was not preceded or followed by a rainfall reduction, and therefore it was simply an enhanced rainfall in the morning rather than a shifting of the diurnal cycle. Figure 3d–f shows the raw counts of increased (+ 1, red) and decreased (− 1, blue) hourly rainfall rates for the month. This analysis was done in order to make sure that the mean values in Fig. 3a–c (magenta) were not impacted by a single event with a large FIRE-NOFIRE difference. Consistently with Fig. 3a–c, there is no clear change in the diurnal cycle of rainfall, whereas the CHEM simulations (cyan markers) show a clear enhancement of rainfall in CHEM-FIRE over Regions 1 and 3 (i.e., over land) in later UTC hours, as pointed out in TW22. Therefore, it is fair to conclude that the WRF simulations do not exhibit the same fire effect as in the CHEM simulation, with respect to the mean, total, and diurnal cycle of the precipitation during the month simulated.

## Discussion

**Figure 4:**
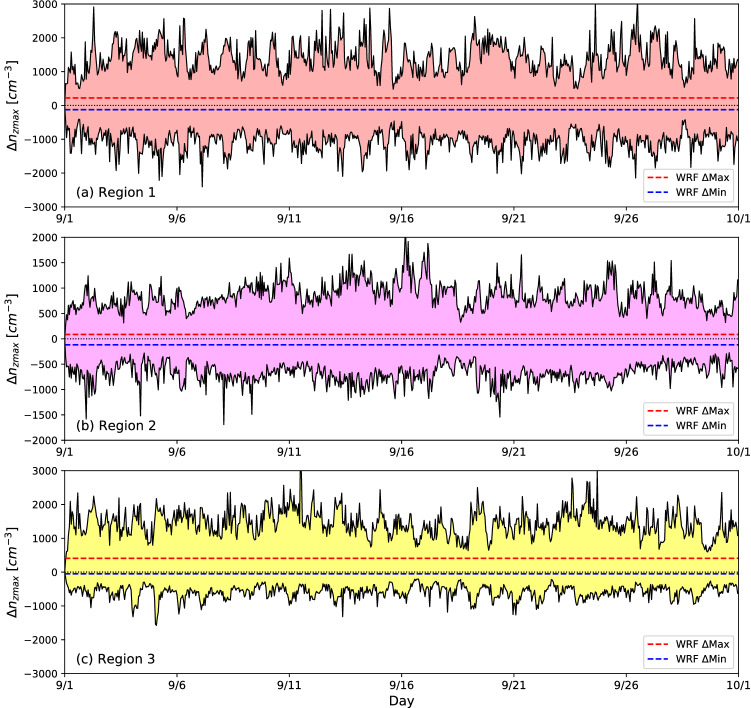
Temporal evolution of maximum and minimum ${\Delta }n_{zmax}$ shown by the shading, in (**a**) Region 1, (**b**) Region 2, and (**c**) Region 3. The maximum and minimum ${\Delta }{\overline{n}}_{zmax}$ are shown in red and blue lines, respectively. Note that ${\Delta }n_{zmax}$ in this figure (shading) was based on columns where there was a cloud (i.e., $q_c\,\ge\,0.001~\text {gm}^{-3}$) in both CHEM-FIRE and CHEM-NOFIRE at each plotted moment, whereas the ${\Delta }{\overline{n}}_{zmax}$ values are the differences in the monthly mean values ${\overline{n}}_{zmax}$ that were separately calculated for CHEM-FIRE and CHEM-NOFIRE regardless of the existence of clouds in the other run. Therefore, the average of ${\Delta }n_{zmax}$ (the upper and lower ends of the shading) in this figure may not exactly correspond to the red and blue lines.

According to TW22, the rainfall differences between CHEM-FIRE and CHEM-NOFIRE largely stemmed from the differences in $n_c$, or the microphysical effect of aerosols. The radiative effects of aerosols actually worked towards weakening convection via surface cooling in CHEM-FIRE, but its strength was small. The WRF simulations do not include the direct radiative effect of aerosols and include only the representation of the microphysical effects. Given the fact that WRF-FIRE and WRF-NOFIRE did not exhibit the same ACI as in the CHEM simulations, it is suggested that not only the spatial but also the *temporal* variations of $n_c$ is indispensable for realizing the ACI similarly to that in a chemistry model. As is discussed in the Method section, the prescribed $n_c$ in WRF-FIRE and WRF-NOFIRE are based on the monthly mean vertical maximum $n_c$ in each column (${\overline{n}}_{zmax}$) in CHEM-FIRE and CHEM-NOFIRE, respectively. The variable $n_{zmax}$ is useful for comparing the WRF and WRF-CHEM runs because it remains constant in the WRF simulations given the temporally invariant $n_c$, whereas it varies both spatially and temporally in the WRF-CHEM simulations. When we make a column-by-column comparison between FIRE and NOFIRE, the differences ${\Delta }n_{zmax}$ are expected to be mostly positive given the general increase of $n_c$ in the FIRE runs (both WRF and WRF-CHEM), but can be negative in columns where the NOFIRE runs had a larger $n_c$ for various reasons; for instance, clouds in CHEM-FIRE may have formed under a very clean condition after a rain event, or more clouds may have formed in an area by chance in CHEM-NOFIRE. In Fig. 4, we show the spatial (vertical extent) and temporal (x axis) variations of ${\Delta }n_{zmax}$ in the three regions. The minimum and maximum ${\Delta }n_{zmax}$ are shown by the blue and red lines for WRF and by the shading for WRF-CHEM. This figure highlights: (1) the large spatial variability of ${\Delta }n_{zmax}$ at each time step that is indicated by the vertical extent of the shaded area, and (2) its large temporal variability. Although ${\overline{n}}_{zmax}$ is also spatially variable as the difference between the blue and red lines shows, the variability is not as large as the instantaneous differences in the CHEM runs (i.e., the vertical extent of the shading) because ${\overline{n}}_{zmax}$ is based on monthly mean $n_{zmax}$. In all the three regions, it is evident that there exists a huge spatial and temporal variability in ${\Delta }n_{zmax}$, which cannot be easily represented by prescribed $n_c$. In order for non-chemistry models to incorporate such temporal and spatial variations in $n_c$, data from a chemistry model can be used to constrain the concentrations, although that may not fully resolve the problem if the resolution of the chemistry model is not as fine as the host model. The use of a chemistry model is therefore still ideal for conducting an ACI study in a region with a wide range of $n_c$ expected over time.

Lastly, since the primary reason for choosing the WRF model over WRF-CHEM is its lower computational expense, here we note the difference in the computational cost upon using the two models. Within 20 h that is the maximum allocated time for a single job using multiple nodes on the supercomputer *Jean-Zay*, WRF-CHEM and WRF were able to advance approximately 3.5 days and 23 days in model time, respectively, when using the same number of computing nodes. The run time of WRF-CHEM was therefore about 6 times longer than that of WRF, and the default file size was about 5 times larger. Even though these specific numbers depend on the simulation configurations, the higher computational cost of WRF-CHEM is apparent. The optimal balance between the accuracy of simulated ACI and the limitations in computational resources needs to be rigorously examined in each ACI study, and moreover, the means to incorporate the temporal variations of $n_c$ at a low computational cost (e.g., machine-learning) needs to be pursued in future ACI studies.

## Methods

We have utilized the WRF model^4^ version 3.6.1 to conduct the month-long WRF simulations (WRF-FIRE and WRF-NOFIRE) presented in this study. The simulation domains, horizontal resolutions, vertical levels, and time steps are all identical to those in the CHEM simulations presented in TW22: the resolution of 20 km (4 km), 50 vertical levels, and the time step of 30 s (6 s) for the parent (nested) domain. The simulations were initialized with the CHEM-NOFIRE simulation at 0000 UTC on September 1, 2015, presented in TW22. The meteorology conditions at the boundaries were provided by the NCEP Final Analysis data (GFS-FNL)^10^, as was the case for the CHEM simulations. Physics and dynamical settings are also identical to those in the CHEM simulations, which are elaborated in TW22. As in the CHEM simulations, the upper limit on ice number concentrations in the original Morrison scheme^11^ was removed. The results presented in this study were based on the simulated data in the nested domain.

**Figure 5:**
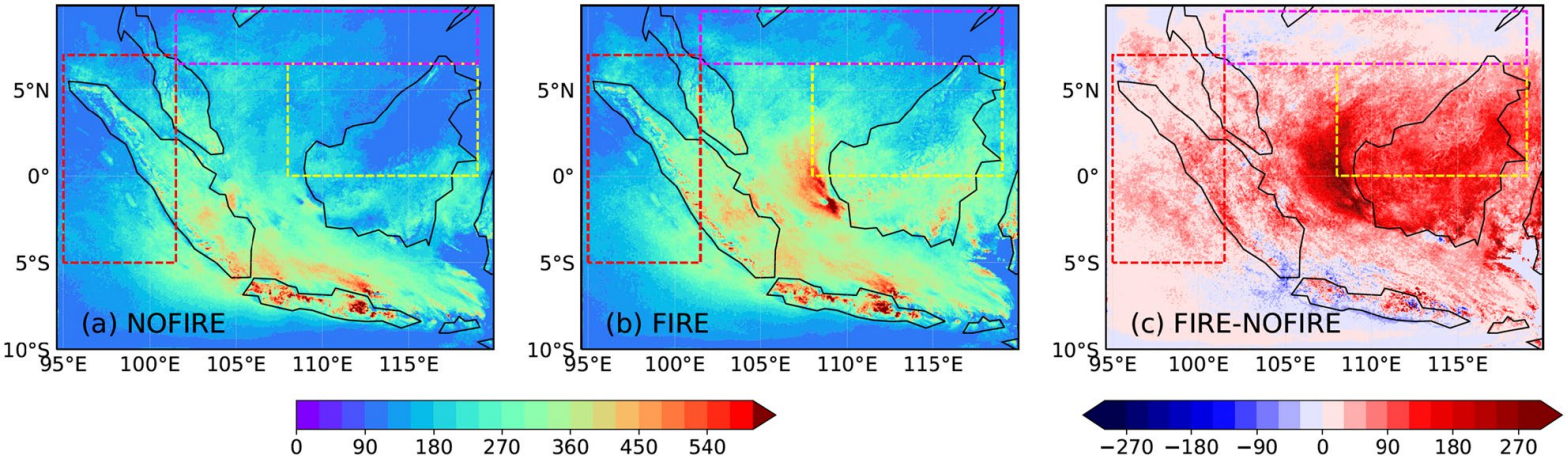
Maps of ${\overline{n}}_{zmax}$ (cm$^{-3}$) for (**a**) WRF-NOFIRE, (**b**) WRF-FIRE, and (**c**) their difference (FIRE-NOFIRE) over the nested domain.

The maps of monthly mean vertical maximum $n_c$ in each column (${\overline{n}}_{zmax}$), used for WRF-NOFIRE and WRF-FIRE simulations, are shown in Fig. 5 and were derived from the CHEM-NOFIRE and CHEM-FIRE simulations as follows; for both the parent and nested domains, a vertical maximum cloud droplet number concentration ${n}_{zmax}$ was calculated for each column every hour for the month of September, only when at least one grid point with a cloud (i.e., cloud mass $q_c \ge 0.001~\text {gm}^{-3}$) existed within the column. The monthly average of these values for each column is ${\overline{n}}_{zmax}$. If the calculated values of ${\overline{n}}_{zmax}$ were lower than 100 cm$^{-3}$, then ${\overline{n}}_{zmax}$ was set to 100 cm$^{-3}$ in those columns. This minimum value was arbitrarily chosen so that extremely low $n_c$ are avoided over the area where few if any clouds were simulated in the CHEM simulations. The two-dimensional values in Fig. 5a,b were read-in by the WRF model so that $n_c$ within each column was always set to the same value in Fig. 5a,b.

## Supplementary Information

Supplementary Information.

## Data Availability

The WRF model is available on the website of the University Corporation for Atmospheric Research (UCAR) at https://www2.mmm.ucar.edu/wrf/users/download/get_source.html. The meteorological input data from GFS-FNL is also available on the UCAR website at https://rda.ucar.edu/datasets/ds083.2/^10^. TRMM data can be found on the website of the National Aeronautics and Space Administration (NASA) at https://disc.gsfc.nasa.gov/datasets/TRMM_3B43_7/summary^7^. The modified source code of the Morrison microphysics scheme and the ${\overline{n}}_{zmax}$ data are available upon request.
