# Supplementary material for: Comparison of the simulated outcomes of aerosol–cloud interaction by a meteorological model with and without an interactive chemistry module
Source: Sci Rep. 2023 Apr 7;13:5694. doi: 10.1038/s41598-023-32355-4 (PMC10082002; doi:10.1038/s41598-023-32355-4)
Supplement: Supplementary file 1 — Supplementary Information. [file 41598_2023_32355_MOESM1_ESM.pdf]

Supplementary Information for "Comparison of the Simulated Outcomes of Aerosol-Cloud Interaction by a Meteorological Model With and Without an Interactive Chemistry Module"

Azusa Takeishi[1,*] and Chien Wang[1]

[1]Laboratoire d'Aérologie, UPS/CNRS, 14 avenue Edouard Belin, Toulouse, 31400, France
[*]azusa.takeishi@aero.obs-mip.fr

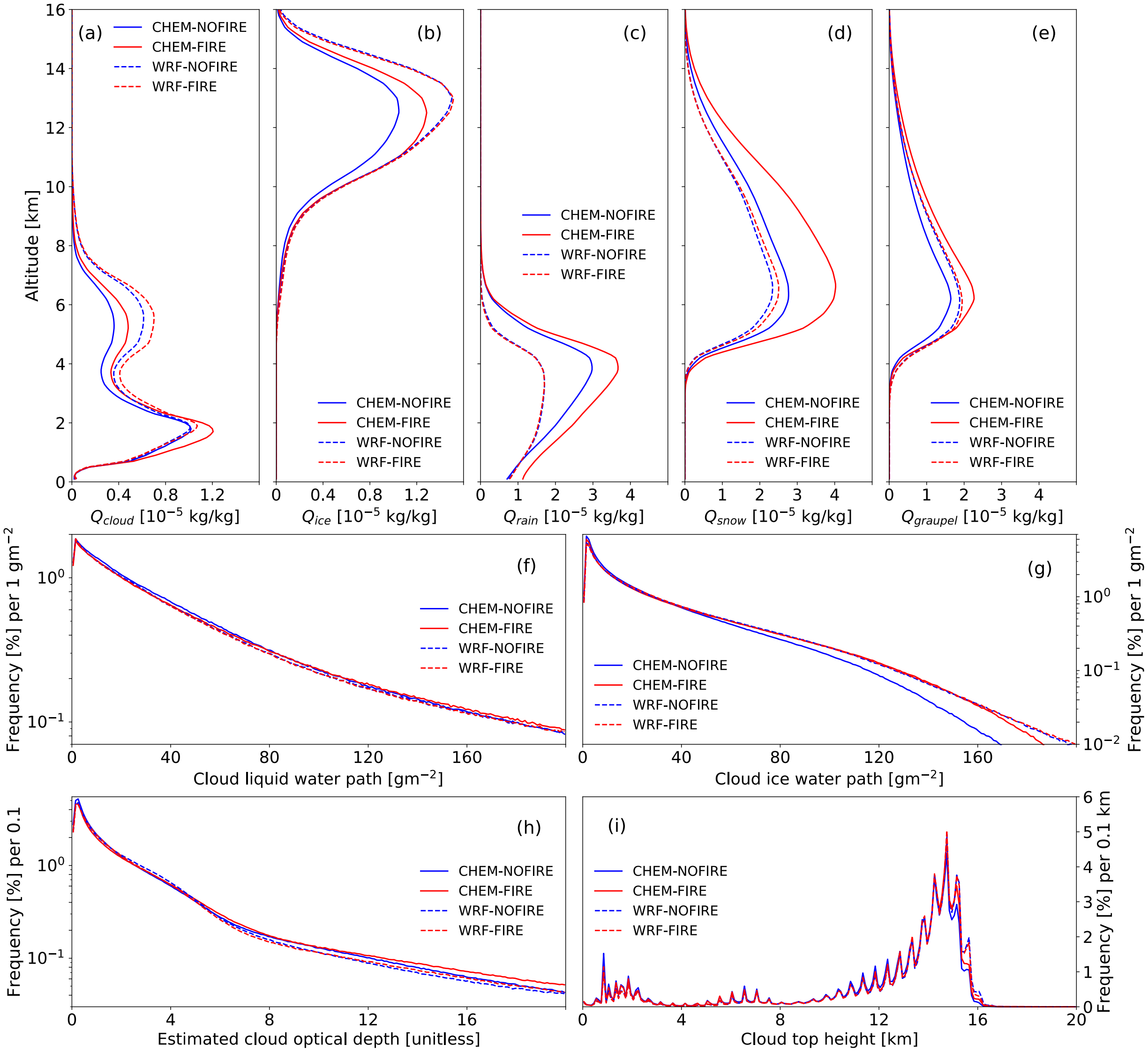

Figure S1. Monthly mean vertical profiles of (a) liquid cloud, (b) ice crystals, (c) rain, (d) snow, (e) and graupel mass mixing ratios [10^{-5} kg/kg] averaged within Region 3. The averaging included all columns within the region. Frequencies of occurrence [%] of cloud liquid water path [gm^{-2}], cloud ice water path [gm^{-2}], estimated cloud optical thickness, and cloud top height [km] are shown in (f)-(i), respectively. These frequencies were calculated for each 1/200 segment of the shown x-axis range. To avoid non-cloudy columns in (f)-(i), only columns with at least one grid point of liquid cloud mass ≥ 10^{-3}gm^{-3}, ice cloud mass ≥ 10^{-3}gm^{-3}, or the sum of liquid and ice cloud mass ≥ 10^{-3}gm^{-3} were used for the calculations in (f), (g), (h-i), respectively. This threshold was chosen based on Lu et al., (2014). The result in (i) is partly limited by the available vertical levels in the simulations.

References

Lu, C., Liu, Y., Niu, S. & Endo, S. Scale dependence of entrainment-mixing mechanisms in cumulus clouds. *J. Geophys. Res. Atmospheres* **119**, 13,877–13,890, DOI: 10.1002/2014JD022265 (2014).
